# How many movements in a scribble? A method for quantifying “continuous” perseveration in cancellation tasks

**DOI:** 10.3389/fnhum.2013.00332

**Published:** 2013-07-02

**Authors:** Alessio Toraldo

**Affiliations:** Department of Brain and Behavioural Sciences, University of PaviaPavia, Italy

## Scribbles in cancellation tasks

Neglect patients often show perseveration while crossing out targets in a paper-and-pencil cancellation task (e.g., Na et al., 1999; Bottini and Toraldo, 2003; Toraldo et al., 2005). Thus they may cancel a target by producing more than one mark, or by carrying out a continuous, uninterrupted movement which produces a *scribble* rather than a well-formed, simple mark (“continuous perseveration,” Sandson and Albert, 1984, 1987). The topic has been debated with growing interest in the last few years, especially regarding the problem of whether perseveration intensity correlates with neglect severity: some authors reported a significant, positive correlation (e.g., Na et al., 1999; Nys et al., 2006), some others failed to find it (e.g., Pia et al., 2009; Ronchi et al., 2009). Settling such an issue would be relevant to decide whether the two deficits share some underlying mechanisms (e.g., Posner et al., 1984; Toraldo et al., 2005), or are functionally independent (e.g., Ronchi et al., 2009). I reasoned that part of the discrepancies between results in the literature might be due to differences in the grain of analysis of perseverative behaviors, with coarse-grained methods failing to detect effects that were instead found by finer-grained methods. Hence in this paper I will propose a fine-grain analysis yielding a very sensitive measure of one specific perseveration type, i.e., scribbling behavior.

The ideal measure of scribbling behavior would be a count of the (linear or circular) movements that were performed to produce the scribble. The use of high-definition cameras for filming the patient's performance (Kim et al., 2009), or of graphic tablets to record it, is not always possible or practical, especially in clinical settings (patients with marked perseverative symptomatology are most typically in the acute phase), and might interfere with natural motor behavior by the patient. Indeed, virtually all studies in the field were retrospective analyses of cancellation marks produced on paper sheets. The purpose of the present work is to develop a retrospective method that, given a scribble, allows one to obtain a proxy of the number *N* of movements that were made to produce it, even though no video recording of the performance is available. This would allow the experimenter to perform a fine-grain analysis of scribbling behavior on much larger patient samples, thus increasing statistical power.

## Logic of the method

**Figure 1:**
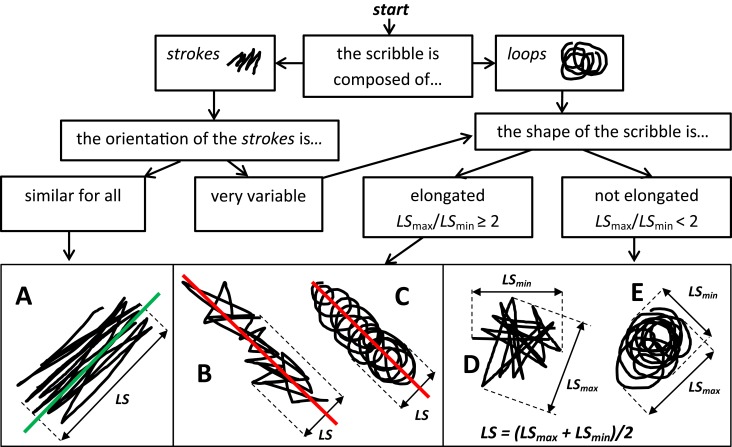
**Flowchart for deciding along what direction the linear size (*LS*) of a scribble should be measured**. (i) If the scribble is composed of (roughly linear) *strokes* that are all oriented in a similar way (as in **A**), the scribble has most likely been produced by a distal oscillatory movement along the direction of the strokes (green line in **A**), combined with a transportation component orthogonal to it; hence *LS* should be measured along the green line (the likely direction of the distal component). (ii) If the scribble is not a set of well-aligned strokes as in **A**, another clue is the scribble's shape: if this is *elongated* (as in **B**,**C**: its maximum linear size *LS_max_* is more than twice its minimum linear size *LS_min_*) the transportation component likely acted along its major axis (red lines); in order to minimize the impact of the transportation component, *LS* will be measured orthogonally to such a direction. (iii) If neither of the above conditions applies (like in **D**,**E**), *LS* can be estimated as the average between the maximum and the minimum *LS* obtained from the scribble (see formula in **D**,**E**). (iv) Clearly the set of five examples (**A–E**) is not exhaustive: there may be scribbles containing both strokes and loops, or having a markedly curvilinear transportation component, etc. In these cases the experimenter can use the rules that apply to the example which most closely resembles the scribble under study; also, the idea that *LS* should be computed orthogonally to the transportation component (**B,C**) can be extended to elongated scribbles having a curvilinear transportation component. Anyway, virtually all of the 206 scribbles that were produced by 33 right hemisphere patients (Gandola et al., 2007) correspond to one of the five examples (mostly, types **A** and **E**).

The basic assumption is that the “atom” of cancellation behavior is a single, elementary movement, which can either be (roughly) unidirectional and linear—leading to the production of what I will call a *stroke*, or (roughly) circular—leading to the production of a shape resembling a full (360°) circle or ellipse, which I will call a *loop*. Thus strokes and loops are assumed to be the elementary units of cancellation behavior, and a scribble is assumed to be the result of a continuous sequence of strokes/loops, made without breaking the pen-to-paper contact; scribbles look quite different according to whether strokes (e.g., Figures 1A,B,D) or loops (Figures 1C,E) compose them.

The purpose of the present work is that of reconstructing the *N* of strokes/loops composing a scribble. Crucially, the absolute length of each stroke/loop is irrelevant, because it just reflects the patient's cancellation style—nothing related to the pathological processes underlying perseverative mechanisms. Hence by this principle, two scribbles which are identical in shape but different in size, must obtain the same score because they contain an identical *N* of strokes/loops. One can now understand that the simple area *A*—the surface covered with ink—of the scribble is not a good proxy for *N*, because it does not depend only on *N*: it also reflects the typical length of the single stroke/loop; furthermore, *A* is a function of the pen-tip thickness, another irrelevant factor which can vary from subject to subject and needs to be partialled out.

I started from the notion that the *N* of strokes/loops composing a scribble equals the total length of the pathway that the pen traced on paper (*L*), divided by the average length of the strokes/loops (*l*). Hence, the proposed proxy for *N* will have the form *L/l*. Of course, the problem with most scribbles is that the extensive overlap between the different strokes/loops makes it impossible to reconstruct the original trajectory of the pen-tip; hence *L* and *l* will be impossible to measure directly. In the next section, indirect estimates of *L* and *l* will be proposed that rely on measures that can be easily obtained.

## Proxies for *L* and *l*

Intuitively, a good proxy for the total pathway length *L* is the scribble's area *A* divided by the thickness *t* of the pen-trace (which can easily be measured by considering an isolated portion of a stroke/loop). So, *A/t* can be used to estimate *L*. The next step is to replace the average length of the strokes/loops, *l*, with a measure which can be easily obtained and is strictly proportional to it, i.e., the scribble's *linear size* (*LS*). Indeed, the longer the average stroke/loop, the larger the scribble will be. This allows one to use *LS* instead of *l*; however this only holds for scribbles composed of strokes (as in Figures 1A,B,D); for scribbles composed of loops (as in Figures 1C,E), the proxy for *l* will be π *LS* (the length of a loop is roughly π times its diameter, which in turn is proportional to *LS*).

Of course, *LS* will, in general, over-estimate *l* (a scribble is wider than the single strokes/loops composing it), and *A/t* will under-estimate the real pathway length *L* (because of the often extensive overlap between different strokes/loops in the scribble); however this is not much of a problem, since we are not searching for unbiased estimates, but for good *proxies*, i.e., measures that have a high correlation with the target of the measurement. Certainly *LS* is highly correlated with *l*, and *A/t* is highly correlated with *L*, so they are good proxies.

## In what direction should the linear size *LS* be measured?

Consider the typical dynamics of the scribbling gesture. Such a motor act is the combination of two components: the shift of the arm-wrist across the sheet—henceforth, the *transportation* or “proximal” component, and the cyclical movements of the hand-fingers producing the strokes/loops—henceforth, the *distal* component. Since we are interested in the size of the strokes/loops (*l*), we should measure *LS* (our proxy for *l*) so that it reflects the distal component of the movement as much as possible, and the transportation component as little as possible. Figure 1 reports some clues that help identifying the likely direction of the two components, in order to be able to measure *LS* along the direction of the distal component. A flowchart is provided with the suggested procedure.

## The *Scribble Perseveration Index* (*SPI*), a proxy for *N* of strokes/loops in the scribble

In summary, *A/t* and *LS* (or π *LS* in the case of loop-composed scribbles) will be used as proxies for *L* and *l* respectively. Actually, the adjusted versions *A/t–t* and *LS–t* will be used to take into account the extreme case in which the pen-tip touched the paper in a single point, without travelling any distance over it (thus, *L* = 0): the produced dot will have *LS* = *t* and *A* = (about) *t*^2^, hence *A/t−t* and *LS*−*t* will correctly be null.

So, the formula *L/l* becomes (*A/t*−*t*)/(*LS*−*t*). This will be the proxy for the *N* of strokes/loops composing a scribble. The minimal number of strokes/loops, of course, is 1. So if one subtracts 1, s/he will obtain a measure expressing the number of strokes/loops exceeding 1, i.e., the number of perseverative strokes/loops:

$$\begin{array}{l} \left[\text{Strokes}\right]\;SPI\text{​}=\text{​}(A/t\text{​}-\text{​}t)/(LS\text{​}-\text{​}t)-1\\ \left[\text{Loops}\right]\;SPI\text{​}=\text{​}(A/t\text{​}-\text{​}t)/[\pi (LS\text{​}-\text{​}t)]-1 \end{array}$$

where *A* = scribble area; *t* = pen-trace thickness; *LS* = scribble linear size (measured as specified in Figure 1). The measurement unit for *A* must be the square of the unit for *t* and *LS*.

The obtained values may, for statistical fluctuations, be negative—since clearly the number of perseverative strokes/loops cannot be less than zero, such scores must be equated to zero.

### Range and meaning of the *Scribble Perseveration Index* (*SPI*)

An *SPI* of 0 indicates a single, perfectly linear stroke, or a perfect, single circle—that is, the total absence of any perseverative tendency. The minimal amount of non-zero scribbling behavior, e.g., two consecutive strokes (like in a V-sign), obtain a positive *SPI* (on average, 0.41). And so on, with the *SPI* increasing with increasing *N* of underlying strokes/loops. Note that the higher *N*, the more *SPI* will under-estimate *N* in absolute terms. Such under-estimation is the effect of using *LS* as a proxy for *l*, and of the fact that, the higher the number of strokes/loops, the more they will overlap with each other (hence yielding an under-estimation of *L*). Thus, e.g., the scribbles in Figures 1A–E, which were produced with, respectively, *N* = 17, 25, 13, 18, and 11 strokes/loops, obtained an *SPI* of 7.6, 15, 6.2, 7.8, and 3 respectively. The amount of *N*-underestimation, which is specific to every individual dataset, was estimated to be 72.6% in the experiment reported in the next section.

## How good a proxy is *SPI* for *N*?

### Method

To estimate the validity of *SPI* as a proxy for the real *N* of strokes/loops, a simple experiment was performed, in which the author produced 96 scribbles according to the following 2 × 2 × 6 design (4 repetitions per cell): (i) using an ordinary pen vs. a thick crayon; (ii) drawing strokes vs. loops; (iii) varying the amount of perseveration across six levels, from no perseveration at all (one single stroke, one single loop) to a massive amount of perseveration, thus covering the whole range of perseverative behaviors that are usually observed in brain-damaged patients (Gandola et al., 2007). The performance was filmed by means of a high-resolution digital camera. The recordings were reproduced in slow-motion (1/8×) in order to accurately count the *N* of strokes/loops made to produce every scribble. The four A4-sheets containing the 96 scribbles were then scanned (100 dpi), TIFF images were obtained, and the areas *A*, pen-trace thicknesses *t*, and linear sizes *LS* were all obtained by using the Open Source software ImageJ (http://rsbweb.nih.gov/ij/). The procedure involved (i) digitalizing the image by using a MaxEntropy threshold =234; (ii) estimating *A* in 100-dpi-pixels (function Histogram); (iii) measuring *LS* in inches/100 (function Measure).

### Results

*N* (obtained from the inspection of the video-recordings) ranged 1–22. *SPI*s were obtained by applying the above formulae, and ranged 0–6.2. The linear regression analysis yielded the following equation: *SPI* = 0.2743 × *N*–0.1403. Most crucially, the Pearson correlation between *N* and *SPI* was *r* = +0.9695. Note that the simple area *A* of the scribble was much worse a proxy for *N*: *r* = +0.4938. Indeed, *A* also reflects differences in movement type (linear, circular), movement amplitude, pen-thickness (ordinary pen vs. crayon), etc.

## Conclusions

I proposed a method which provides a proxy for the number of movements (linear or circular) that were carried out to produce a scribble. The measures needed for computing the *Scribble Perseveration Index* (*SPI*) are easy to obtain by means of many graphic computer programs (e.g., ImageJ), and are: (i) the scribble's area, (ii) the scribble's linear size, and (iii) the thickness of the pen-trace. *SPI* proved to be a very good proxy for *N*: the correlation between them was +0.9695. Clearly this validity value is to be considered as an upper limit—it has been obtained from a single subject (albeit the scribbles that were used closely resemble the vast majority of scribbles that can be observed in right hemisphere patients: Gandola et al., 2007). Such an upper limit might be increased in the future, e.g., by inserting adjustment terms in the formulae which take into account the degree of overlap between different strokes/loops in the scribble, a factor which is likely to vary greatly across scribbles (and across patients). Nonetheless, a validity value of 0.97 is high by any standard, so that *SPI* can be proposed as a tool for in-depth analyses of “continuous” perseveration (Sandson and Albert, 1984, 1987) in cancellation tasks performed by brain-damaged patients. I hope *SPI* will help addressing some of the open questions in neglect research, e.g., the puzzling problem of whether neglect severity and perseveration intensity correlate (see section Scribbles in Cancellation Tasks).
